# Circulating cell-free mitochondrial DNA levels in Parkinson’s disease are influenced by treatment

**DOI:** 10.1186/s13024-020-00362-y

**Published:** 2020-02-18

**Authors:** Hannah Lowes, Angela Pyle, Mauro Santibanez-Koref, Gavin Hudson

**Affiliations:** 1 Wellcome Centre for Mitochondrial Research, Biosciences Institute, Newcastle University, Newcastle upon Tyne, NE1 3BZ UK; 2 Wellcome Centre for Mitochondrial Research, Translational and Clinical Research Institute, Newcastle University, Newcastle upon Tyne, NE2 4HH UK; 3 Biosciences Institute, Newcastle University, Newcastle upon Tyne, NE1 3BZ UK

**Keywords:** Parkinson’s disease, Circulating cell-free mitochondrial DNA, Biomarker, Neurodegeneration

## Abstract

Several studies have linked circulating cell-free mitochondrial DNA (ccf-mtDNA) to human disease. In particular, reduced ccf-mtDNA levels in the cerebrospinal fluid (CSF) of both Alzheimer’s and Parkinson’s disease (PD) patients have raised the hypothesis that ccf-mtDNA could be used as a biomarker for neurodegenerative disease onset and progression. However, how a reduction of CSF ccf-mtDNA levels relates to neurodegeneration remains unclear. Many factors are likely to influence ccf-mtDNA levels, such as concomitant therapeutic treatment and comorbidities. In this study we aimed to investigate these factors, quantifying CSF ccf-mtDNA from the Parkinson’s Progression Markers Initiative in 372 PD patients and 159 matched controls at two time points. We found that ccf-mtDNA levels appear significantly reduced in PD cases when compared to matched controls and are associated with cognitive impairment. However, our data indicate that this reduction in ccf-mtDNA is also associated with the commencement, type and duration of treatment. Additionally, we found that ccf-mtDNA levels are associated with comorbidities such as depression and insomnia, however this was only significant if measured in the absence of treatment. We conclude that in PD, similar to reports in HIV and sepsis, comorbidities and treatment can both influence ccf-mtDNA homeostasis, raising the possibility that ccf-mtDNA may be useful as a biomarker for treatment response or the development of secondary phenotypes. Given that, clinically, PD manifests often decades after neurodegeneration begins, predicting who will develop disease is important. Also, identifying patients who will respond to existing treatments or develop secondary phenotypes will have increased clinical importance as PD incidence rises.

## Main text

The identification of circulating cell-free mitochondrial DNA (ccf-mtDNA) in 2000 [1], prompted a wave of studies assessing the utility of ccf-mtDNA as a biomarker of disease [2–7], detection of cancers [8–12] and susceptibility to comorbidities during HIV infection [13, 14].

Ccf-mtDNA is thought to be released as a by-product of cell death [15, 16] or, in response to increased oxidative or metabolic stress as a damage-associated molecular pattern [17]. Thus, it might be expected that ccf-mtDNA would be elevated in disease. However, the opposite appears true in neurodegenerative disease, with studies showing reduced cerebrospinal fluid (CSF) ccf-mtDNA in Alzheimer’s (AD) [18] and Parkinson’s disease (PD) [19] patients; which decreases further during disease course. This suggests that reduced ccf-mtDNA could be a biomarker of both disease onset and progression; however, what this reduction reflects biologically and how it relates to neurodegeneration remains unclear.

Many factors likely influence ccf-mtDNA levels. Disease is clearly a factor^2–713,14^, raising the possibility that comorbidities may modulate PD ccf-mtDNA levels. Serum ccf-mtDNA levels are influenced by inflammation [20], BMI [21] and psychosocial and physical stress [22]; factors previously linked to PD progression [23, 24]. Elevated plasma ccf-mtDNA levels are associated with nonresponsiveness to selective serotonin reuptake inhibitor (SSRI) treatment in major depressive disorder (MDD) [4] and vitamin C infusion has been shown to reduce plasma ccf-mtDNA levels in sepsis patients [25], suggesting that treatment may be an important cofactor in any ccf-mtDNA assessment. In CSF, ccf-mtDNA likely originates from the ependymal cells of the choroid plexus, an area not subject to neurodegeneration in either AD or PD, but which recruits leukocytes to the brain under inflammatory conditions [26]. CSF ccf-mtDNA was reduced in multiple sclerosis (MS) patients who were treated with fingolimod [27], a drug which inhibits lymphocyte release from lymph nodes and reduces autoreactive inflammation in the central nervous system. Fingolimod also suppresses neuronal mitochondrial mediated autophagy [28], suggesting that disease modulatory treatments may influence CSF ccf-mtDNA levels by suppressing mtDNA release during cellular stress.

In PD, L-dopa reportedly exacerbates neuroinflammation [29] and inflammation is associated with ccf-mtDNA release [30]. Therefore, it is possible that L-dopa treatment could increase ccf-mtDNA levels in responders, but have no effect in those that have become L-dopa resistant. In addition, L-dopa may affect the mtDNA pool available for release, as L-dopa can induce oxidative stress [31], affecting mitochondrial vitality and increasing cellular mtDNA copy-number in brains [32].

However, despite evidence suggesting that variable ccf-mtDNA levels could be a manifestation of treatment [25], this has not been formally tested in PD. Thus, in this study we assessed CSF ccf-mtDNA levels in a well-characterised cohort of PD subjects and controls to test the relationship between treatment and changes in ccf-mtDNA.

PD and control CSF samples, biopsied at 0 and 36 months, were obtained from the Parkinson’s Progression Markers Initiative (PPMI). Ccf-mtDNA was quantified by qPCR [19, 33]. Statistical analysis of log [10] transformed ccf-mtDNA levels were performed using Student’s t-test, ANOVA and ANCOVA, with effect sizes estimated using Cohen’s d. Detailed protocols are available in the Methods Section.

PPMI-PD patients and controls were well-matched (Additional File 1**:** Table 1). As expected, PPMI-PD patients had significantly higher clinical severity ratings when compared to controls at both 0 and 36 months. At 0 months no PPMI-PD case was receiving PD-related medication. By 36 months 90% had begun treatment; with 25% receiving L-dopa only, 6% receiving dopamine agonists only, 6% receiving monoamine oxidase inhibitors only and 63% receiving a combination of at least two of these treatments (Additional File 1**:** Table 1).

**Fig. 1:**
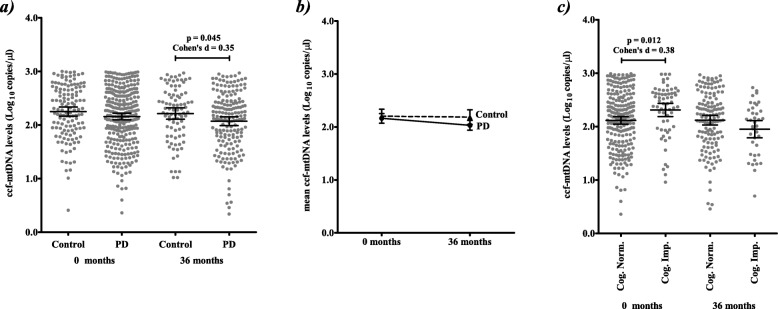
Comparative ccf-mtDNA levels in PPMI-PD cases and controls. **a** CSF ccf-mtDNA levels are reduced in PPMI-PD patients compared to controls at 36 months (mean ccf-mtDNA in 176 PD patients 2.0 (95%CI = 1.99–2.16), versus 2.2 (95%CI = 2.11–2.32) in 87 controls, Student’s t-test *p* = 0.045, Cohen’s d = 0.35), but not at 0 months (mean ccf-mtDNA in 291 PD patients 2.1 (95%CI = 2.10–2.22), versus 2.2 (95%CI = 2.16–2.34) in 132 controls, Student’s t-test *p* = 0.101). After regression, adjusting for age, sex and BMI as covariates, group differences remained significant at 36 months (*p* = 0.047) and non-significant at 0 months (*p* = 0.233). **b** The reduction in CSF ccf-mtDNA between 0 to 36 months appears specific to PPMI-PD cases (Change in mean CSF ccf-mtDNA levels calculated from 0 to 36 months where both timepoints were available, mean change in 130 PD patients − 0.15 (95%CI = 0.274--0.026), Student’s t-test p = 0.045; in 58 controls − 0.02 (95%CI = -0.224–0.184), Student’s t-test *p* = 0.858). **c** Elevated CSF ccf-mtDNA levels are associated with cognitive impairment in PPMI-PD patients at 0 months (mean ccf-mtDNA in 230 PD cases without cognitive impairment 2.1 (95%CI = 2.05–2.19), versus 2.3 (95%CI = 2.19–2.45) in 61 PD cases with cognitive impairment, Student’s t-test *p* = 0.012, Cohen’s d = 0.38), but not at 36 months (mean ccf-mtDNA in 138 PD cases without cognitive impairment 2.1 (95%CI = 2.03–2.21), versus 1.9 (95%CI = 1.79–2.11) in 38 PD cases with cognitive impairment, Student’s t-test *p* = 0.44). Error bars indicate mean and 95% confidence interval (95%CI)

At recruitment (0 months) ccf-mtDNA levels were not significantly different between PPMI-PD cases and controls, however after 36 months ccf-mtDNA levels appeared significantly reduced in PPMI-PD cases, although with a modest effect size (Fig. 1a). Limiting to samples with both 0 and 36 month measurements confirms that this reduction is limited to PD cases (Fig. 1b**;** individual changes in ccf-mtDNA copy number between 0 and 36 months are presented in Additional File 1**: **Fig. 1); in line with our previous report [19]. Within PPMI-PD subjects, we found no significant correlations between disease severity ratings or motor-related phenotypes and ccf-mtDNA levels at 0 or 36 months (Additional File 1**: **Table 2). Elevated ccf-mtDNA was associated with cognitive impairment (defined by MoCA < 26 and psychometric testing defined by the PPMI) at 0 months (Fig. 1c); however, this trend was reversed at 36 months. Previous studies have used the Unified Parkinson’s Disease Rating Scale (UPDRS) to measure the response of motor and non-motor symptoms to treatment [34]. However, comparing change in 36 month ccf-mtDNA levels to changes in UPDRS parts I-III and UPDRS-total score (calculated as 36 months minus the month at which PD treatment began) failed to identify a significant association (data not shown). Although, as PPMI-PD UPDRS scores have been shown to significantly increase over time [35], this may be a reflection of the reduced sample number with a viable CSF ccf-mtDNA measurement (i.e. 48% of the samples used in [35]) and variability in treatment durations (average treatment duration prior to 36 month sampling in 158 treated PD cases was 26.8 ± 5.9 months); suggesting that further, longitudinal, studies are required.

**Fig. 2:**
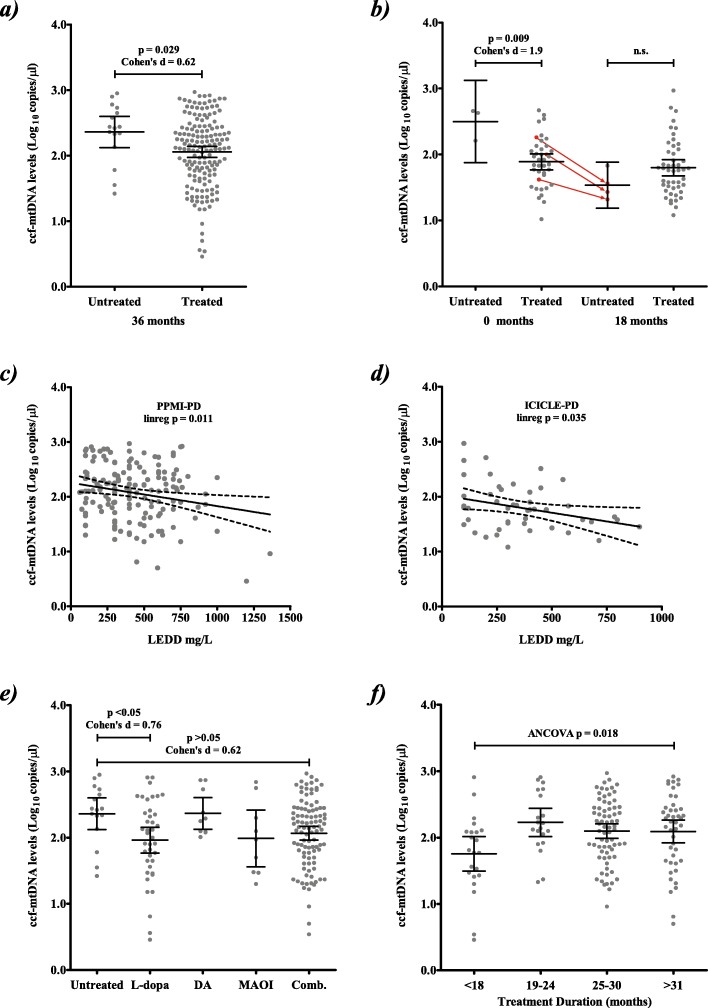
Ccf-mtDNA levels associate with PD treatment**. a** CSF ccf-mtDNA levels in treated PD patients are significantly lower than untreated patients (mean ccf-mtDNA in 160 treated PPMI-PD patients 2.1 (95%CI = 1.97–2.14) versus 2.4 (95%CI = 2.12–2.60) in 16 untreated PPMI-PD patients, Student’s t-test *p* = 0.029, Cohens d = 0.62). **b** A reanalysis of our previously published data [19], with ICICLE-PD patients now grouped as treated/untreated further supports our findings that treatment affects ccf-mtDNA levels at 0 months (mean ccf-mtDNA in 39 treated ICICLE-PD patients 1.9 (95%CI = 1.77–2.01), versus 2.5 (95%CI = 1.88–3.13) in 3 untreated ICICLE-PD patients, Student’s t-test *p* = 0.009, Cohens d = 1.9). At 18 months, ccf-mtDNA levels appeared similar between untreated/treated PD patients (*n* = 4/48, *p* = 0.36). However, 3 out of the 4 18-month untreated cases were receiving treatment at 0 months (indicated by the arrows), suggesting that the effect of treatment on reducing ccf-mtDNA had already occurred, although this is based on a small subset and should be interpreted with caution. **c-d** CSF ccf-mtDNA levels are significantly inversely correlated to levodopa effective daily dose (LEDD) in **c)** PPMI-PD (*n* = 157, 36 month samples, *p* = 0.011, r2 = 0.05) and **d** ICICLE-PD (*n* = 48, 18 month samples, *p* = 0.035, r2 = 0.1) patients. Dotted lines indicate 95% CI. **e** Specific treatment type is significantly associated to CSF ccf-mtDNA levels (ANOVA *p* = 0.048), with levodopa/carbidopa treated PPMI-PD patients showing significantly reduced ccf-mtDNA levels compared to untreated patients (mean ccf-mtDNA 2.0 (95%CI = 1.77–2.16) in 40 levodopa/carbidopa treated PD versus 2.4 (95%CI = 2.12–2.60) in 16 untreated PD, Dunnett’s *p* < 0.05, Cohens d = 0.76). CSF ccf-mtDNA levels appear lower in patients treated with combination therapies, although this did not reach statistical significance (Dunnett’s *p* > 0.05). Where L-dopa = levodopa/carbidopa, DA = dopamine agonist, MAOI = monoamine oxidase inhibitor and Comb = combination of > 2 treatments. **f)** CSF ccf-mtDNA levels are associated to treatment duration (18 to > 31 months ANCOVA *p* = 0.018, with treatment type as a covariate), with PPMI-PD patients treated for < 18 months showing a significant reduction in CSF ccf-mtDNA levels when compared to each subsequent time point (Tukey’s corrected *P* < 0.05 for each time point). Error bars indicate 95% confidence interval (95%CI)

Next we investigated the effect of treatment on ccf-mtDNA levels. PPMI-PD patients began treatment in the intervening period between baseline and 36 month, (Additional File 1**:** Table 1**)**, thus we limited our analysis to 36 month data. Although the number of PPMI-PD patients who remained treatment naïve was comparatively small, we observed a significant inverse correlation between treatment and ccf-mtDNA levels (Fig. 2a). This effect was replicated by a reanalysis of our previously published ICICLE-PD data [19], which also showed significantly reduced ccf-mtDNA levels in treated patients (Fig. 2b & Additional File 1: Table 3). To further support this finding, we compared ccf-mtDNA levels to levodopa equivalent daily dose (LEDD) [36], which showed significant inverse correlations in both PPMI-PD and ICICLE-PD separately (Fig. 2c-d), and when analysed together (*p* = 0.0096, Additional File 1**:** Figure 2). Taken together, these data suggest that therapeutic intervention is modulating ccf-mtDNA levels.

**Fig. 3:**
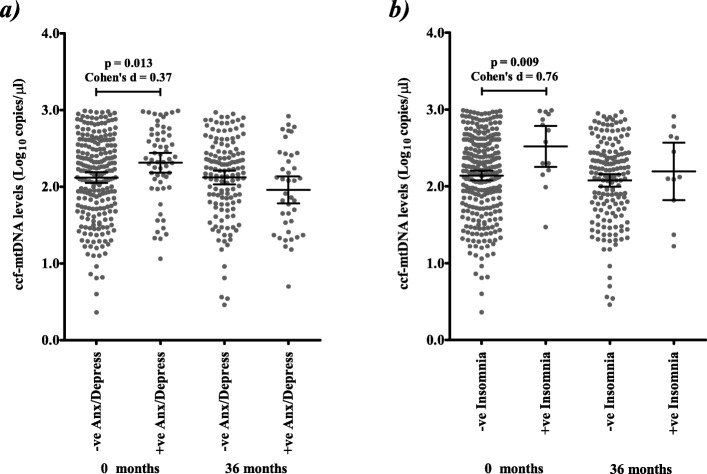
Prior to treatment, ccf-mtDNA levels are associated with PD-related comorbidities**. a** CSF ccf-mtDNA levels are associated with anxiety/depression in PD patients at 0 months (mean ccf-mtDNA in 232 PD cases without anxiety/depression 2.1 (95%CI = 2.05–2.19), versus 2.3 (95%CI = 2.19–2.44) in 59 PD cases with anxiety/depression, Student’s t-test *p* = 0.013, Cohen’s d = 0.37), but not at 36 months (mean ccf-mtDNA in 136 PD cases without anxiety/depression 2.12 (95%CI = 2.03–2.21), versus 1.94 (95%CI = 1.78–2.14) in 40 PD cases with anxiety/depression, Student’s t-test *p* = 0.092). **b** CSF ccf-mtDNA levels are associated with insomnia in PD patients at 0 months (mean ccf-mtDNA in 277 PD cases without insomnia 2.1 (95%CI = 2.08–2.20), versus 2.5 (95%CI = 2.25–2.79) in 14 PD cases with insomnia, Student’s t-test p = 0.009, Cohen’s d = 0.76), but not at 36 months (mean ccf-mtDNA in 165 PD cases without insomnia 2.1 (95%CI = 2.00–2.79), versus 2.2 (95%CI = 1.82–2.57) in 11 PD cases with insomnia, Student’s t-test *p* = 0.486). Error bars indicate 95% confidence interval (95%CI)

Sub-stratification of PPMI-PD data by treatment type indicates that L-dopa is significantly inversely associated with ccf-mtDNA levels (Fig. 2e). Although combination therapies (of which L-dopa is a component) showed the same trend, this did not reach statistical significance in PPMI-PD. The effect of L-dopa was replicated in the ICICLE-PD data, with combination therapy also reaching statistical significance (Dunnett’s *p* < 0.05, Additional File 1**: **Figure 3), although it should be noted that the group sizes are comparatively smaller.

We then compared treatment duration to ccf-mtDNA levels by ANCOVA, which showed a significant association when analysed with treatment type as a covariate (*p* = 0.018, Fig. 2f). Patients on treatment for < 18 months had significantly reduced ccf-mtDNA levels compared to subsequent timepoints (Tukey’s p < 0.05, Fig. 2f), with ccf-mtDNA levels rising as treatment duration increased, suggesting that treatment has an immediate effect that diminishes over time. This effect was not treatment type specific, with each of the four treatments showing reduced ccf-mtDNA at 18 months, subsequently rising with duration (Additional File 1**:**Figure 4). Those ICICLE-PD patients on treatment, were on treatment at both 0 and 18 months and were thus not used to assess treatment time.

Several phenotypes are associated with ccf-mtDNA [2–7, 9–14, 37] and PD is associated with a number of comorbidities [38]. Thus, we stratified ccf-mtDNA levels by PD-related comorbidities including: anxiety/depression (anxiety and depression were 99.9% concordant in PD patients) [39], gastroesophageal reflux disease [40], constipation [41], insomnia [42], diabetes [43] and sleep apnoea [44] (Additional File 1**:** Table 4). At 0 months, we observed significantly elevated ccf-mtDNA levels in PD patients exhibiting anxiety/depression (*p* = 0.013, Fig. 3a) and PD patients exhibiting insomnia (*p* = 0.009, Fig. 3b). However, by 36 months (and, notably, after the initiation of PD treatment) the trend is reversed for anxiety/depression (Figs. 3a**,** Additional File 1**:** Table 4), but not for insomnia (although the sample size is smaller); further suggesting that therapeutic intervention is the principle driver of changes in ccf-mtDNA reduction.

Our results show that ccf-mtDNA levels are reduced in PD patients compared to controls and, similar to other studies [4, 13, 14], can be indicative of comorbidities. However, this reduction is also influenced by the commencement, type and duration of therapeutic intervention.

Elevated ccf-mtDNA was observed in MDD patients who were unresponsive to SSRI treatment [4] and reduced ccf-mtDNA is associated with vitamin C infusion in sepsis patients [25], suggesting that ccf-mtDNA levels may be an indicator of treatment response. With regard to PD medication, L-dopa crosses the blood-brain barrier and has been shown to affect mitochondrial function [45, 46], increasing neuronal mtDNA copy-number [47]. Thus, if we assume that ccf-mtDNA release is the norm (based on observations that the levels in controls are often higher than patients) [19, 48], it is possible that the initial decrease in ccf-mtDNA we observe in patients taking L-dopa (and L-dopa in combination treatments) may actually be a consequence of the drive to increase cellular mtDNA content by restricting the fraction of mtDNA that is released. As L-dopa becomes less effective as PD progresses [49], the subsequent increase in ccf-mtDNA we observe over time is potentially a result of selection of the surviving neurons or a result of a proportion of patients becoming unresponsive to continued treatment.

Both PD, L-dopa and ccf-mtDNA are linked to inflammation [20, 50], however, we were unable to directly measure this in our cohort. This is an important area of future study, as we are unaware of previous reports which have compared CSF ccf-mtDNA levels to inflammation in PD. Further, ccf-mtDNA was measured in CSF which, although often used for studying markers of neurodegeneration [51], cannot be wholly indicative of localised differences in cell death or mitochondrial dysfunction. Thus, in the future it may be advisable to correlate CSF ccf-mtDNA levels to specific brain pathology and brain mtDNA levels, to potentially elucidate the biological mechanisms underlying ccf-mtDNA release.

Finally, our data suggest that ccf-mtDNA levels may be associated with the onset of comorbidities such as cognitive impairment, anxiety/depression, and insomnia, but only if measured in the absence of treatment; suggesting that the effect of treatment on reducing ccf-mtDNA is greater than the effect of comorbidities to raise it. It is possible that there is an interplay between ccf-mtDNA, PD medication and the response to additional medications given to treat each comorbidity, however, we were unable to assess in this study. Thus, independent replication of these observations is warranted and future ccf-mtDNA studies should consider these factors as potential confounding variables.

In conclusion, our results indicate that ccf-mtDNA levels can be influenced by treatment commencement, type and duration; which limits the utility of ccf-mtDNA as a biomarker of disease onset. In addition, our observations that LEDD dose correlates to ccf-mtDNA level is worthy of further mechanistic investigation. Our data also indicate that, considering inconsistencies in reported disease associations to ccf-mtDNA, studies of ccf-mtDNA where treatment parameters and comorbidity have been omitted or were unavailable should be interpreted with caution.

## Methods section

### Protocol approvals, registrations, and consents

Written informed consent for research was obtained from all individuals participating in the PPMI and approved by an ethical standards committee (PPMI-info.org).

### PPMI CSF sample cohort

We used 541 PPMI lumbar CSF biopsies, sampled at 0 months (372 PD patients and 169 matched-controls), and 364 PPMI lumbar CSF biopsies, sampled at 36 months (250 PPMI-PD cases and 114 matched-controls). Sample drop-out between 0 months and 36 months was 33% (33% cases and 33% controls). For all samples, CSF was collected and stored under PPMI guidance (fully described at https://www.ppmi-info.org/). Demographic data is summarised in Additional File 1: Table 1.

### PPMI cohort characteristics

At 0 months, all PPMI-PD patients had a clinical diagnosis of PD for < 2 years and were not taking PD medication (although a large proportion, 90.3%, of patients had begun treatment at 36 months, Additional File 1: Table 1.**:** with 25.2% of PD patients receiving levodopa(+carbidopa) only, 6.22% receiving dopamine agonists only, 5.6% receiving monoamine oxidase inhibitors only and 62.9% receiving a combination of at least two of these treatments). All control participants were > 30 years of age, did not have PD or prodromal signs of PD and did not have a first degree blood relative with PD (fully described at https://www.ppmi-info.org/). Demographic and phenotypic data at 0 months and 36 months were made available from the PPMI (fully described at https://www.ppmi-info.org/). Phenotypic data is summarised in Additional File 1: Table 1.

### Ccf-mtDNA quantification

ccf-mtDNA was quantified using established methods [19, 33]. ccf-mtDNA copy number was calculated as an absolute measurement of *MTND1* (minor deletion arc mitochondrial gene) and derived from a triplicated standard curve and is expressed as copies per 1 μl of CSF. As in previous work [19, 33], samples with > 1 *B2M* copies per microliter (indicating nuclear DNA contamination) were removed from further analysis (0 months: PD patients = 81, 28% and controls = 37, 28%, and 36 months: PD patients =74, 30% and controls = 27, 23%), leaving a final cohort of 423 samples at 0 months (291 PD patients and 132 controls) and 263 samples at 36 months (176 PD patients and 87 controls).

### Statistical analysis

Data were analysed using R (v3.4.3) [52] and Prism v5. Normality of ccf-mtDNA distributions were assessed by Shapiro-Wilks and could not be rejected at the 0.05 level. Thus, all ccf-mtDNA levels are expressed as log [10] copy-number per microliter.

Correlations were performed using Pearson’s r, control vs PD comparisons were performed using Student’s t-tests, while comparisons of treatment type and duration were performed using ANOVA (with Dunnett’s post-hoc tests, using the first category as a reference, i.e. untreated in Fig. 2e) or by ANCOVA with treatment type as a covariate (with Tukey’s post-hoc tests, i.e. in Fig. 2f). All tests were two-tailed with α =0.05. Multiple significance correction can be too conservative for a discovery study, particularly when testing a priori hypotheses with variables that are not all independent [53]. Thus, unless specified in the text, we report unadjusted *P* values as for reasons well documented in the literature [53].

### ICICLE-PD reanalysis

In previous work [19], we observed a significant reduction in ccf-mtDNA levels at both 0 and 18 months. As the vast majority (> 95%) were on treatment at study commencement, we did not originally consider the effect of treatment. In this subsequent reanalysis we have revisited our original data, taking treatment commencement and type into account (Additional File 1: Table 3). Ccf-mtDNA data and treatment were reanalysed as per PPMI-PD.

### Statistical power

Based on prior association [19], where mean log [10] ccf-mtDNA per microliter was 1.8 (SD = 0.48) in 54 PD compared to 2.4 (SD = 0.32) in 10 controls, and assuming an α of 5%, we have > 95% power to detect a significant difference in mean ccf-mtDNA copy number between PD patients and controls using Student’s t-test at baseline assuming > 50 cases versus > 50 controls (calculated using pwr v1.2–2 in R).

## Supplementary information

**Additional file 1 Table 1.** Demographic and clinical charateristics of PD patients and controls at baseline (0 months) and 36 months. **Table 2.** Comparison of ccf-mtDNA levels to clinical severity ratings and PD-related phenotypes at 0- and 36-months. **Table 3.** Frequency of ICICLE-PD patient treatment at 0 and 18 months. **Table 4.** Comparsion of ccf-mtDNA levels between PD-related comorbidities at 0- and 36-months. **Figure 1.** Individual changes in ccf-mtDNA copy number, from 0 to 36 months. **Figure 2.** Correlation of ccf-mtDNA levels to LEDD for merged PPMI-PD and ICICLE-PD data. **Figure 3.** Ccf-mtDNA levels associate with PD specific treatments in ICICLE-PD. **Figure 4.** Linear regression of mean ccf-mtDNA levels versus treatment duration for each PD treatment

## Data Availability

The dataset generated and analysed during this study is available in the Parkinson’s Progression Markers Initiative repository upon request (https://www.ppmi-info.org/access-data-specimens/download-data/).

## Abbreviations

AD: Alzheimer’s Disease
BMI: Body mass index
ccf-mtDNA: Circulating cell free mitochondrial DNA
CI: Confidence interval
Comb: Combination of > 2 treatments (i.e. DA & MAOI)
CSF: Cerebrospinal fluid
DA: Dopamine agonist
L-dopa: Levodopa/carbidopa
MAOI: Monoamine oxidase inhibitor
MS: Multiple sclerosis
PD: Parkinson’s disease
UPDRS: Unified Parkinson’s disease rating scale
