# Supplementary material for: Circulating cell-free mitochondrial DNA levels in Parkinson’s disease are influenced by treatment
Source: Mol Neurodegener. 2020 Feb 18;15:10. doi: 10.1186/s13024-020-00362-y (PMC7029508; doi:10.1186/s13024-020-00362-y)
Supplement: Supplementary file 1 — Additional file 1 Table 1. Demographic and clinical charateristics of PD patients and controls at baseline (0 months) and 36 months. Table 2. Comparison of ccf-mtDNA levels to clinical severity ratings and PD-related phenotypes at 0- and 36-months. Table 3. Frequency of ICICLE-PD patient treatment at 0 and 18 months. Table 4. Comparsion of ccf-mtDNA levels between PD-related comorbidities at 0- and 36-months. Figure 1. Individual changes in ccf-mtDNA copy number, from 0 to 36 months. Figure 2. Correlation of ccf-mtDNA levels to LEDD for merged PPMI-PD and ICICLE-PD data. Figure 3. Ccf-mtDNA levels associate with PD specific treatments in ICICLE-PD. Figure 4. Linear regression of mean ccf-mtDNA levels versus treatment duration for each PD treatment [file 13024_2020_362_MOESM1_ESM.docx]

***Additional File***

*Contents:*

- *Table 1. Demographic and clinical charateristics of PD patients and controls at baseline (0 months) and 36 months.*
- *Figure 1.* *Individual changes in ccf-mtDNA copy number, from 0 to 36 months.*
- *Table 2.* *Comparison of ccf-mtDNA levels to clinical severity ratings and PD-related phenotypes at 0- and 36-months.*
- *Table 3. Frequency of ICICLE-PD patient treatment at 0 and 18 months.*
- *Figure 2. Correlation of ccf-mtDNA levels to LEDD for merged PPMI-PD and ICICLE-PD data.*
- *Figure 3. Ccf-mtDNA levels associate with PD specific treatments in ICICLE-PD.*
- *Figure 4. Linear regression of mean ccf-mtDNA levels versus treatment duration for each PD treatment.*
- *Table 4. Comparsion of ccf-mtDNA levels between PD-related comorbidities at 0- and 36-months.*

**Table 1. Demographic and clinical charateristics of PPMI-PD patients and controls at baseline (0 months) and 36 months.**

The table shows the frequency or mean (with 95% confidence interval) of each characteristic in PPMI-PD samples. Where p-value is a comparison by Student’s t-test, p-values are unadjusted (**Detailed Methods Section** and^51^)

|  | ***Baseline (0 months)*** | | | ***36 months*** | | |
| --- | --- | --- | --- | --- | --- | --- |
|  | ***PD patients (291)*** | ***Controls (132)*** | ***p-value*** | ***PD patients (176)*** | ***Controls (87)*** | ***p-value*** |
| *Ethnicity:*  *% White*  *% Hispanic*  *% Indian*  *% Asian*  *% Black*  *% Not Specified* | *90.7% (264)*  *2.7% (8)*  *1.0% (3)*  *3.1% (9)*  *1.7% (5)*  *0.7% (2)* | *93.2% (123)*  *1.5% (2)*  *0.0% (0)*  *0.8% (1)*  *4.5% (6)*  *0.0% (0)* | *n.s.*  *n.s.*  *n.s.*  *n.s.*  *n.s.*  *n.s.* | *92.0% (162)*  *3.4% (6)*  *1.1% (2)*  *1.1% (2)*  *1.7% (3)*  *0.6% (1)* | *93.1% (81)*  *2.3% (2)*  *0.0% (0)*  *1.1% (1)*  *3.4% (3)*  *0.4% (1)* | *n.s.*  *n.s.*  *n.s.*  *n.s.*  *n.s.*  *n.s.* |
| *Age (at CSF sample)* | *61.2* *(60.2-62.3)* | *59.9 (58.0-61.9)* | *0.039* | *63.6* *(62.2-65.0)* | *64.6* *(62.2-66.9)* | *n.s.* |
| *% Sex (m/f)* | *66%/34%* | *62%/38%* | *n.s.* | *66%/34%* | *63%/37%* | *n.s.* |
| *Age of Onset (years)* | *60.1* *(59.6-61.8)* | *-* | *-* | *60.0 (58.6-61.5)* | *-* | *-* |
| *Disease Duration (months)* | *6.80* *(6.0-7.5)* | *-* | *-* | *44.8 (43.0-46.5)* | *-* | *-* |
| *Mean UPDRS-I* | *2.1* *(2.0-2.3)* | *1.6* *(1.3-1.6)* | *<0.001* | *3.1* *(2.7-3.4)* | *1.5* *(1.3-1.7)* | *<0.001* |
| *Mean UPDRS-II* | *6.9* *(6.4-7.3)* | *1.6* *(1.4-1.8)* | *<0.001* | *10.1* *(9.2-11.0)* | *1.5* *(1.3-1.6)* | *<0.001* |
| *Mean UPDRS-III* | *21.3* *(20.2-22.4)* | *1.1* *(2.2-3.6)* | *<0.001* | *28.1* *(26.25-29.97)* | *1.7* *(2.4-4.2)* | *<0.001* |
| *Mean UPDRS-V (H&Y)* | *1.6* *(1.5-1.6)* | *1.0* *(1.0)* | *<0.001* | *1.8* *(1.8-1.9)* | *0.0* | *<0.001* |
| *Mean Total UPDRS score* | *30.3* *(28.9-31.7)* | *4.1* *(3.7-4.7)* | *<0.001* | *42.2* *(39.7-44.8)* | *4.7* *(4.0-5.4)* | *<0.001* |
| *Mean MoCA score^(1)^* | *27.1* *(26.9-27.4)* | *28.1* *(28.0-28.4)* | *<0.001* | *26.5* *(26.1-26.9)* | *27.5* *(27.0-28.0)* | *0.004* |
| *% on PD Treatment* | *0%* | *-* | *-* | *90.9% (160)* | *-* | *-* |
| *Where received L-dopa* | *0%* | *-* | *-* | *25.2% (40)* | *-* | *-* |
| *Where received DA* | *0%* | *-* | *-* | *6.3% (10)* | *-* | *-* |
| *Where received MAOI* | *0%* | *-* | *-* | *5.6% (9)* | *-* | *-* |
| *Where received combination^(2)^* | *0%* | *-* | *-* | *62.9% (100)* | *-* | *-* |
| *Where PD treatment <18m* | *0%* | *-* | *-* | *13.9% (22)* | *-* | *-* |
| *Where PD treatment 19-24m* | *0%* | *-* | *-* | *12.7% (20)* | *-* | *-* |
| *Where PD treatment 25-30m* | *0%* | *-* | *-* | *45.6% (72)* | *-* | *-* |
| *Where PD treatment >31m* | *0%* | *-* | *-* | *27.8% (44)* | *-* | *-* |

*UPDRS, unified Parkinson's disease rating scale; MoCA, Montreal cognitive assessment. L-dopa - levodopa or L-3,4-dihydroxyphenylalanine (in combination with carbidopa), DA - dopamine agonist, MAOI - Monoamine oxidase inhibitor. Showing mean and 95% CI (x-x). ^(1)^0-month MoCA was assessed 45 days prior to baseline. ^(2)^Combination treatment was >=2 of the other treatments (i.e. L-Dopa and MAOIs).*

**AF Figure 1. Individual changes in CSF ccf-mtDNA copy number, from 0 to 36 months for Parkinson’s disease (PD) patients and controls.** PD in red, controls in blue.

**Table 2. Comparison of ccf-mtDNA levels to PPMI-PD clinical severity ratings and PD-related phenotypes at 0 and 36 months.**

The table shows the frequency of each characteristic in PPMI-PD cases, mean log(10) ccf-mtDNA and 95% confidence interval. Where *is comparison by Student’s t-test of mean ccf-mtDNA and **is correlation of ccf-mtDNA by Pearson’s correlation coefficient. UPDRS, unified Parkinson's disease rating scale. P values are unadjusted (**Detailed Methods Section** and^51^)

|  |  |  | ***0-Month PD (n=291)*** | | |  | ***36-Month PD (n=176)*** | | |
| --- | --- | --- | --- | --- | --- | --- | --- | --- | --- |
|  |  |  | ***% Freq.***  ***(Count)*** | ***Mean***  ***ccf-mtDNA*** | ***p-value*** |  | ***% Freq.***  ***(Count)*** | ***Mean***  ***ccf-mtDNA*** | ***p-value*** |
| ***Clinical severity Score*** | *UPDRS-I*** | | *100 % (291)* | *-* | *0.235* |  | *100% (176)* | *-* | *0.805* |
|  | *UPDRS-II*** | | *100 % (291)* | *-* | *0.983* |  | *100% (176)* | *-* | *0.485* |
|  | *UPDRS-III*** | | *100 % (291)* | *-* | *0.643* |  | *100% (176)* | *-* | *0.372* |
|  | *UPDRS-V*  *(H&Y)*** | | *100 % (291)* | *-* | *0.574* |  | *100% (176)* | *-* | *0.231* |
|  | *Total UPDRS*** | | *100 % (291)* | *-* | *0.609* |  | *100% (176)* | *-* | *0.587* |
|  |  |  |  |  |  |  |  |  |  |
| ***Motor Diagnosis*** | *with*  *Tremor** | *Y* | *79% (229)* | *2.15* *(2.1-2.2)* | *0.959* |  | *77% (136)* | *2.04 (2.0-2.1)* | *0.116* |
|  |  | *N* | *21% (62)* | *2.16 (2.0-2.3)* |  |  | *23% (40)* | *2.19 (2.1-2.3)* |  |
|  | *with*  *Rigidity** | *Y* | *72% (209)* | *2.14 (2.1-2.2)* | *0.639* |  | *75% (132)* | *2.09 (2.0-2.2)* | *0.297* |
|  |  | *N* | *28% (82)* | *2.18 (2.1-2.3)* |  |  | *25% (44)* | *1.99 (1.9-2.2)* |  |
|  | *with*  *Bradykinesia** | *Y* | *83% (242)* | *2.13 (2.1-2.2)* | *0.075* |  | *83% (146)* | *2.07 (2.0-2.2)* | *0.994* |
|  |  | *N* | *17% (49)* | *2.28 (2.2-2.5)* |  |  | *17% (30)* | *2.07 (1.9-2.3)* |  |
|  | *with*  *Postural Inst.** | *Y* | *6% (18)* | *2.14 (1.9-2.4)* | *0.873* |  | *8% (14)* | *2.01 (1.7-2.3)* | *0.667* |
|  |  | *N* | *94% (273)* | *2.16 (2.1-2.2)* |  |  | *92% (162)* | *2.08 (2.0-2.2)* |  |
|  |  |  |  |  |  |  |  |  |  |
| ***Cognitive Phenotype*** | *with*  *Cognitive Impairment** | *Y* | *21% (61)* | *2.31 (2.2-2.4)* | ***0.012*** |  | *22% (38)* | *1.94 (1.9-2.2)* | ***0.440*** |
|  |  | *N* | *79% (230)* | *2.12 (2.1-2.2)* |  |  | *78% (138)* | *2.11 (2.1-2.2)* |  |

**Table 3. Frequency of ICICLE-PD patient treatment at 0 and 18 months.**

The table shows the percentage of ICICLE-PD patients on treatment when CSF sampling was conducted (at 0 months and 18 months) and ccf-mtDNA data was available. The table also shows the breakdown of treatment type, similar to the PPMI-PD cohort (**Table 1**).

|  | ***0 months*** | ***18 months*** |
| --- | --- | --- |
|  | ***PD patients (42)*** | ***PD patients (52)*** |
| ***% on PD Treatment*** | ***39 (92%)*** | ***48 (92%)*** |
| *Where received L-dopa* | *6 (15%)* | *3 (6%)* |
| *Where received DA* | *10 (26%)* | *6 (13%)* |
| *Where received MAOI* | *16 (41%)* | *17 (35%)* |
| *Where received combination^(1)^* | *7 (19%)* | *22 (46%)* |

*L-dopa - levodopa or L-3,4-dihydroxyphenylalanine (in combination with carbidopa), DA - dopamine agonist, MAOI - Monoamine oxidase inhibitor.^(1)^Combination treatment was >=2 of the other treatments (i.e. L-Dopa and MAOIs).*

**AF Figure 2. Correlation of ccf-mtDNA levels to LEDD for merged PPMI-PD and ICICLE-PD data.**

CSF ccf-mtDNA levels are significantly inversely correlated to levodopa effective daily dose (LEDD) when PPMI-PD and ICICLE-PD patient data (Figures 2c-d) are merged (n=205 18+36 month samples, p=0.0096, r2=0.05). Dotted lines indicate 95% CI.

**AF Figure 3. Ccf-mtDNA levels associate with PD specific treatments in ICICLE-PD.**

Similar to Figure 2e, L-dopa and combination treatments associate with reduced ccf-mtDNA in ICICLE-PD (ANOVA p=0.0267), with levodopa/carbidopa (n=6) or combination (n=7) treated ICICLE-PD patients showing significantly) reduced ccf-mtDNA levels at 0 months (Dunnett’s corrected p<0.05 versus 3 untreated patients). Note that n=3 (of 4) 18 month untreated cases were receiving treatment at 0 months, suggesting that the effect of treatment on ccf-mtDNA has already occurred in these individuals. Where L-dopa=levodopa/carbidopa, DA=dopamine agonist, MAOI=monoamine oxidase inhibitor and Comb=combination of >2 treatments. Error bars indicate 95% confidence interval.

**AF Figure 4. Linear regression of mean ccf-mtDNA levels versus treatment duration for each PD treatment.**

The slopes are not significantly different (p=0.176), pooled slope p = 0.033. The intercepts are not significantly different (p=0.403), pooled intercept =1.304

**Table 4.** Comparsion of ccf-mtDNA levels between PD-related comorbidities (anxiety/depression (anxiety and depression were 99.9% concordant in PD patients), gastroesophageal reflux disease (GERD), constipation, insomnia, diabetes and sleep apnoea) at 0 and 36 months. The table shows the frequency of each comorbidity in PD cases, mean log(10) ccf-mtDNA and 95% CI. Comparsions were made by Student’s t-test, P values are unadjusted (**Detailed Methods Section** and^51^)

|  |  | ***0-Month PD (n=291)*** | | |  | ***36-Month PD (n=176)*** | | |
| --- | --- | --- | --- | --- | --- | --- | --- | --- |
| ***Versus***  ***ccf-mtDNA level*** |  | ***% Freq.***  ***(Count)*** | ***Mean***  ***ccf-mtDNA*** | ***p-value*** |  | ***% Freq.***  ***(Count)*** | ***Mean***  ***ccf-mtDNA*** | ***p-value*** |
| ***Anxiety/***  ***Depression*** | ***Y*** | ***20.3% (59)*** | ***2.3 (2.2-2.5)*** | ***0.013*** |  | ***22.7% (40)*** | ***1.9 (1.8-2.1)*** | ***0.092*** |
|  | ***N*** | ***79.7% (232)*** | ***2.1 (2.1-2.2)*** |  |  | ***77.3% (136)*** | ***2.1 (2.0-2.2)*** |  |
| *GERD* | *Y* | *8.2% (24)* | *2.1 (1.8-2.3)* | *0.254* |  | *8.5% (15)* | *2.0 (1.8-2.3)* | *0.715* |
|  | *N* | *91.8% (267)* | *2.2 (2.1-2.2)* |  |  | *91.5% (161)* | *2.1 (2.0-2.2)* |  |
| *Constipation* | *Y* | *7.6% (22)* | *2.1 (1.9-2.3)* | *0.436* |  | *10.2% (18)* | *2.0 (1.7-2.3)* | *0.623* |
|  | *N* | *92.4% (269)* | *2.2 (2.1-2.3)* |  |  | *89.8% (166)* | *2.1 (2.0-2.2)* |  |
| ***Insomnia*** | ***Y*** | ***4.8% (14)*** | ***2.5 (2.3-2.8)*** | ***0.009*** |  | *6.3% (11)* | *2.2 (1.8-2.6)* | *0.486* |
|  | ***N*** | ***95.2% (277)*** | ***2.1 (2.1-2.2)*** |  |  | *93.7% (165)* | *2.1 (2.0-2.2)* |  |
| *Diabetes* | *Y* | *4.8% (14)* | *2.3 (2.0-2.6)* | *0.368* |  | *6.3% (11)* | *2.2 (1.9-2.5)* | *0.324* |
|  | *N* | *95.2% (277)* | *2.2 (2.1-2.2)* |  |  | *93.7% (165)* | *2.1 (2.0-2.2)* |  |
| *Sleep*  *Apnoea* | *Y* | *4.8% (14)* | *2.2 (1.9-2.4)* | *0.982* |  | *6.3% (11)* | *1.9 (1.6-2.2)* | *0.263* |
|  | *N* | *95.2% (277)* | *2.2 (2.1-2.2)* |  |  | *93.7% (165)* | *2.1 (2.0-2.2)* |  |
